# Effects of recombinant human interleukin-10 on Treg cells, IL-10 and TGF-β in transplantation of rabbit skin

**DOI:** 10.3892/mmr.2013.1817

**Published:** 2013-11-20

**Authors:** KAI SHAN LIU, XIAO QIN FAN, LEI ZHANG, QIONG NA WEN, JI HONG FENG, FU CHAO CHEN, JUN MIN LUO, WAN BANG SUN

**Affiliations:** 1 The Border Armed Police Central Hospital of Guangdong Province, Shenzhen, Guangdong 518023, P.R. China; 2 College of Life Science and Teaching, Jinan University, Guangzhou, Guangdong 510632, P.R. China; 3 Department of Basic Teaching, Zunyi Medical College, Zhuhai Campus, Zhuhai, Guizhou 519041, P.R. China; 4 Department of Respiratory Diseases, Central People’s Hospital of Zhanjiang, Zhanjiang, Guangdong 524037, P.R. China; 5 Department of Surgical Laboratory, Zunyi Medical College Zhuhai Campus, Zhuhai, Guizhou 519041, P.R. China; 6 Department of Immunology, Institute of Basic Medical Science, Zunyi Medical College, Zunyi, Guizhou 563003, P.R. China

**Keywords:** cytokines, graft rejection, interleukin-10, Treg cells

## Abstract

The current study aimed to investigate the rejection and survival time of grafted skin, and the changes of Treg cells, interleukin 10 (IL-10) and transforming growth factor-β (TGF-β) in peripheral blood following skin transplantation with recombinant human interleukin-10 (rhIL-10) or cyclosporin A (CsA), as well as the role of IL-10 in immunological rejection mechanisms. A total of 36 rabbits were divided into two groups. The skin of a donor rabbit was transplanted onto the back of one receptor rabbit. Receptors were randomly divided into six groups, including rhIL-10 low-dose (5 μg/kg/d), rhIL-10 high-dose (10 μg/kg/d), CsA low-dose (5 mg/kg/d), CsA high-dose (10 mg/kg/d), rhIL-10 (5 μg/kg/d) and CsA (5 mg/kg/d) and negative control normal saline (NS; 1 ml/d). All groups received intramuscular drug injection for ten days, beginning one day prior to skin transplantation surgery. Following transplantation, each rabbit’s peripheral blood was collected at different times. The changes of CD4^+^CD25^+^ regulatory T cells, IL-10 and TGF-β were determined by flow cytometry and enzyme-linked immunosorbent assay. When compared with the control group, the rejection and survival times of the experimental groups were longer following skin graft. Compared with the two CsA groups and the control group, the proportion of CD4^+^CD25^+^ regulatory T cells of rhIL-10 groups was significantly upregulated on the 4th and 7th days following surgery. However, TGF-β levels were not significantly different. Data suggested that the concentration of IL-10 was positively correlated with the proportion of CD4^+^CD25^+^ regulatory T cells. In addition, IL-10 may delay the rejection time of rabbit skin transplantation and prolong the survival time. Thus, the role of IL-10 in inhibited allograft rejection may be associated with CD4^+^CD25^+^ regulatory T cells and IL-10, and may be independent of TGF-β.

## Introduction

Interleukin 10 (IL-10) is known to be a cytokine synthesis inhibitory factor, which functions in inhibiting immunocyte stimulation. A previous study has shown that IL-10 inhibits the synthesis and activity of Th1 and the expression of major histocompatibility complex-II (MHC-II) by decreaseing antigen presentation ability and the activtiy of natural killer cells (1). On the basis of the rejection capability of IL-10, numerous studies have been performed in order to develop a novel immunosuppressive drug for clinical use. The CD4^+^CD25^+^ regulatory T cell (CD4^+^CD25^+^ Treg) is a T cell that exhibits an immunosuppressive function (2,3) and may be resistant to T cell differentiation, enhancement and activity, and thus, is hypothesized to maintain the immunological balance of organisms. Numerous autoimmune diseases may be induced by the quantity decrease and the dysfunction of Tregs. Moreover, Tregs are important in inflammatory diseases, neoplasms and organ transplantation (4). Transforming growth factor-β (TGF-β) exhibits various biological effects including cell differentiation, proliferation and apoptosis, and regulating the growth, development, injuries and regeneration of extracellular materials in many physiological and pathological processes. The current study used allograft rabbit skin transplantation and cyclosporin A (CsA), as a positive control, to observe the rejection time and survival time of grafted skin. The alteration of CD4^+^CD25^+^ regulatory T cells and the levels of IL-10 and TGF-β in the peripheral blood were also investigated. The role of IL-10 in immunological rejection mechanisms was also investigated. The findings may be useful for future research and development of IL-10.

## Materials and methods

### Animals

A total of 36 New-Zealand white rabbits (n=36, male or female) used for the study were aged between 3 and 4 months, weighed between 2 and 2.5 kg, and were obtained from Zunyi Medical College (Zhuhai, China). The study was approved by the Ethics Committee of Zunyi Medical College (Zhuhai Campus, Zhuhai, China). The rabbits were divided into two groups, donors (n=18) and receptors (n=18), for skin transplant surgery. The groups were then divided into six subgroups, with each subgroup containing three rabbits, including normal saline (NS; 1 ml/d), recombinant human interleukin-10 (rhIL-10) low-dose (5 μg/kg/d), rhIL-10 high-dose (10 μg/kg/d), CsA low-dose (5 mg/kg/d), CsA high-dose (10 mg/kg/d) and rhIL-10 (5 μg/kg/d) + CsA (5 mg/kg/d) groups. All rabbits received intramuscular drug injection for ten days, beginning one day prior to skin transplantation surgery.

### Reagents and instruments

The rhIL-10 was expressed by the SMD1168/pPICZaA-hIL-10 engineering strain, which was constructed by the Immunology Department of Zunyi Medical College (Zunyi, China) (5). Anti-rabbit CD4-fluorescein isothiocyanate (FITC) and rabbit CD25 purified antibodies were purchased from Antigenix America Inc. (Melville, NY, USA). Zenon R-Phycoerythrin mouse IgG Labeling kit was purchased from Invitrogen Life Technologies (Carlsbad, CA, USA). Erythrocyte lysis buffer was purchased from BD Biosciences (San Jose, CA, USA). Rabbit IL-10 and rabbit TGF-β detection kits were purchased from Jingtian Biotechnology Company (Beijing, China). A FacsCalibur flow cytometer was purchased from BD Biosciences. The ELx-800 microplate reader was purchased from Bio-Tek (Winooski, VT, USA).

### Skin allograft surgery

Prior to skin allograft surgery, the hair of rabbits was removed from the backs with electric clippers and a neck strap was marked for each animal. The rabbits were anesthetized with intravenous injection of pentobarbital (3%, 0.04 mg/kg). Medium-thickness skin (1.5×1.5 cm) was harvested from the donor and immersed in a penicillin normal saline solution with excess fat tissue removed and then transplanted on to the same area of the receptor. The four corners were coaptated and then bandaged with aseptic vaseline gauze (Sanwell Product Co. Ltd., Beijing China).

### Serum preparation

The peripheral blood was harvested from the ear marginal vein. The blood was collected one day prior to surgery and subsequently, one, four, seven, 14, 21 and 28 days following surgery. Blood samples were allowed to stand at room temperature for 30 min and centrifuged at 716 × g for 20 min to collect serum. Serum IL-10 and TGF-β levels were detected by enzyme-linked immunosorbent assay (ELISA) according to the manufacturer’s instructions (Jingtian Biotechnology Company).

### Flow cytometric analysis (FCM)

The proportion of CD4^+^CD25^+^ Tregs/CD4^+^ T cells was analyzed by FCM. Peripheral blood cell preparations (50 μl) were stained with 5 μl anti-rabbit CD4-FITC and 5 μl anti-rabbit CD25-PE, and incubated at room temperature for 30 min in the dark. Erythrocyte lysis buffer (2 ml) was mixed briefly with a vortex mixer (Mylab Corporation, Beijing, China), incubated at room temperature for 10 min in the dark, centrifuged at 179 × g for 10 min to collect the precipitate. The precipitate was then washed with phosphate-buffered saline (PBS), fixed with 2 ml 1% paraformaldehyde and measured with FCM.

### Statistical analysis

All data underwent statistical analysis using the Statistical Product and Service Solutions version 15.0 (SPSS, Inc., Chicago, IL, USA) and all values are expressed as the mean ± SD. Differences between groups were assessed by the analysis of variance. P<0.05 was considered to indicate a statistically significant difference.

## Results

### Evaluation of allograft survival and rejection time

**Figure 1:**
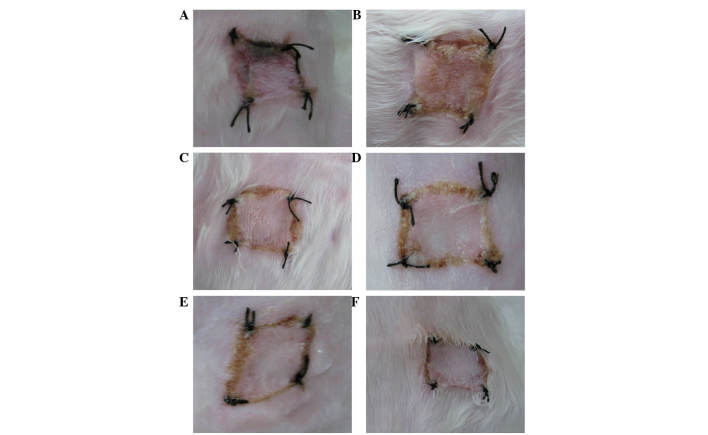
A skin allograft in all groups at day four post-transplantation. (A) The color of the skin graft darkened and exhibited red swelling. (B) The skin graft exhibited red swelling. (C) The skin graft remained close to the wound with little red swelling. (D) The skin graft remained close to the wound with little red swelling. (E) The skin graft remained close to the wound with no secretion. (F) The skin graft remained close to the wound with no secretion.

**Figure 2:**
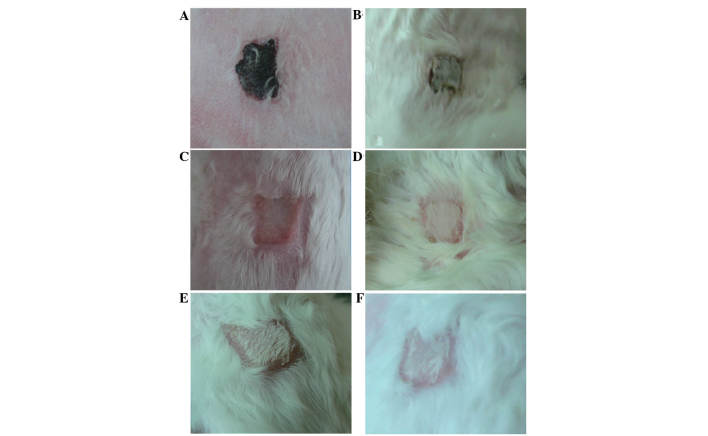
A skin allograft in all groups at day 10 post-transplantation. (A) The entire area of the skin graft darkened with evidence of necrosis. (B) Partial area of the skin graft darkened. (C) The skin graft exhibited red swelling and blistered. (D) The appearance of the allograft with partial new hair growth. (E) The appearance of the allograft with new hair growth is marked compared with (D). (F) The skin graft exhibited partial new hair growth.

**Figure 3:**
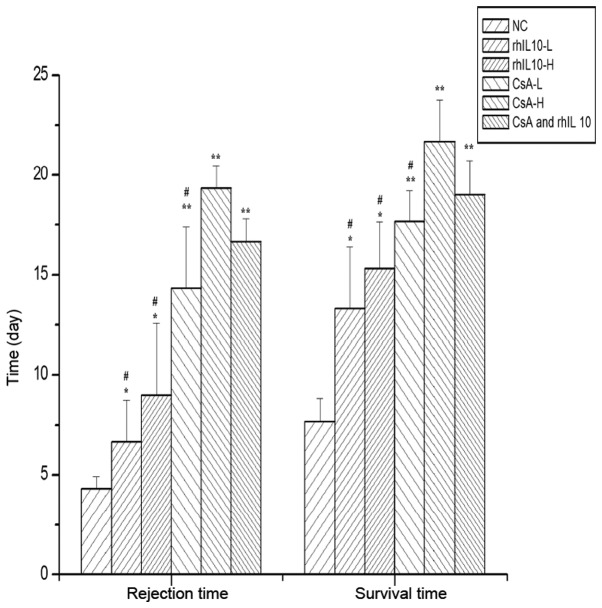
Rejection time and survival time of grafted skin in each group. Compared with the negative control group, the rejection phenomenon and the survival time were significantly longer in the rhIL-10-L, rhIL-10-H, CsA-L, CsA-H, CsA and rhIL-10. Values are presented as the mean ± SEM of each group (^*^P<0.05 and ^**^P<0.01). Compared with the combined medication group the rejection phenomenon and the survival time were significantly lower in the rhIL-10 and CsA low dose group (^#^P<0.05). No significant difference was identified in the combined medication group and CsA high dose group. rhIL-10, recombinant human interleukin-10; CsA, cyclosporin A.

Studies to evaluate allograft survival and rejection time comparing the rejection of the color of the skin graft were preformed. When the color of the skin graft and the skin of the receptor was similar, with no marked inflammation or hyperemia, the skin graft remained in close proximity to the wound and exhibited sufficient drying elasticity with a lack of secretions, which indicated that there was no rejection phenomenon. When the color of the grafted skin was dark, black or swelling was identified, this indicated transplant rejection, and if 80% of the area of the grafted skin appeared as such, it was considered to be necrotic skin according to previous studies (6,7) (Figs. 1 and 2). The allograft survival and rejection time was then determined (Fig. 3). The rejection phenomenon of the treatment groups were slower compared with the control group and the survival time was significantly longer than in the control group (P<0.05). The survival time of the combined medication group was longer than the rhIL-10 and CsA low dose group (P<0.05); however, the survival time between the combined medication group and CsA high dose group was similar (P>0.05).

### rhIL-10 significantly increases the proportion of CD4^+^CD25^+^ Treg/CD4^+^ T cells (%)

**Figure 4:**
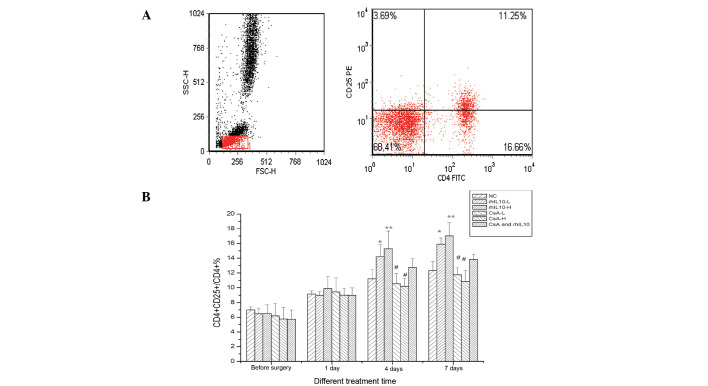
rhIL-10 increases the proportion of CD4^+^CD25^+^/CD4^+^ T cells (%) in peripheral blood. (A) Flow cytometry determined the proportion of CD4^+^CD25^+^ T cells in the CD4^+^ regated T cells (B) Graphical representations of the percentages of CD4^+^CD25^+^/CD4^+^ T cell (%) in peripheral blood at different times. Compared with the levels prior to surgery, the levels of CD4^+^CD25^+^/CD4^+^ T cell (%) in each group were significantly increased. Values are presented as the mean ± SEM of each group. However, the rhIL-10-L and rhIL 10-H groups at days four and seven post-transplantation were markedly higher compared with the NS, CsA-L, CsA-H (^*^P<0.05 and ^**^P<0.01), while the CsA-L, CsA-H was lower compared with NS (^#^P<0.05). rhIL-10, recombinant human interleukin-10; CsA, cyclosporin A; NS, normal saline.

CD4^+^CD25^+^ Treg cells, which constitute ~5–10% of peripheral CD4^+^ T cells, are key in the maintenance of immunological selftolerance and immune responses (8). To determine whether the rhIL-10 had an effect on the proportion of CD4^+^CD25^+^ Treg/CD4^+^ T cells (%), studies were performed to determine the proportion of CD4^+^CD25^+^ Treg/CD4^+^ T cells (%) in each group. As shown in Fig. 4, post-transplantation, the levels of CD4^+^CD25^+^ Treg cells was significantly increased compared with pre-transplantation samples (P<0.05). However, the lower and higher dose groups of rhIL-10 four to seven days following surgery were markedly higher compared with the control and the CsA groups (P<0.05). The lower and higher dose groups of CsA were lower compared with the control group (P<0.05). Thus, rhIL-10 may result in the increased proportion of CD4^+^CD25^+^ Treg/CD4^+^ T cells (%).

### Effects of rhIL-10 on IL-10 and TGF-β levels

**Figure 5:**
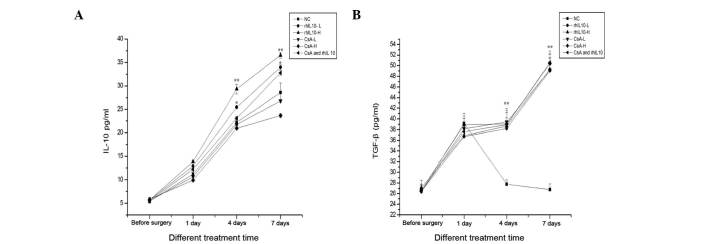
The effects of rhIL-10 on the levels of IL-10 and TGF-β in peripheral blood at different time in each group. The levels of (A) IL-10 and (B) TGF-β were detected by ELISA compared prior to surgery and were significantly increased. However, the rhIL-10-L and rhIL 10-H groups at day 4 and 7 post-transplantation were markedly higher compared with the NS, CsA-L, CsA-H (^*^P<0.05, ^**^P<0.01), while the CsA-L, CsA-H was lower than NS (^#^P<0.05). Compared with NS, the levels of TGF-β at day 4 and 7 post-transplantation was significantly higher (^**^P<0.05). However, no significant difference in each group at days 1, 4 and 7 post-transplantation, compared with the NS was observed. rhIL-10, recombinant human interleukin-10; CsA, cyclosporin A; NS, normal saline.

The effect of rhIL-10 on cytokine expression was investigated. To assess changes in the expression, the levels of IL-10 and TGF-β peripheral blood serum were detected by ELISA. The results are shown in Fig. 5. Post-transplantation, the levels of IL-10 were significantly higher than those pre-transplantation (P<0.05). The lower and higher dose groups of rhIL-10 following surgery between days four and seven were markedly higher compared with the other groups (P<0.05). The lower and higher dose groups of CsA following surgery were significantly lower compared with the rhIL-10 and the combined group. The levels of TGF-β were significantly higher compared with those of preoperative samples (P<0.05). Notably however, no difference was observed among treatment groups following surgery on the 1st, 4th, 7th and 14th day (P>0.05). Thus, rhIL-10 significantly increased the level of IL-10 but not TGF-β.

### Correlation analysis of IL-10 and CD4^+^CD25^+^ Treg/CD4^+^T cells

**Figure 6:**
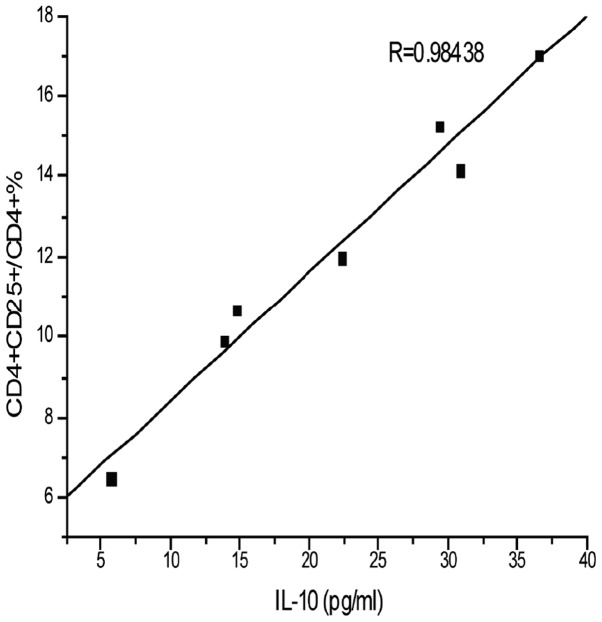
Correlation analysis of CD4^+^CD25^+^ Treg/CD4^+^ T cells (%) and IL-10. IL-10 and the CD4^+^CD25^+^Treg/CD4^+^ T cells (%) were positively correlated. The correlation coefficient R=0.98438. IL-10, interleukin-10.

The aforementioned results were used to further determine the correlation between IL-10 and CD4^+^CD25^+^ Treg/CD4^+^ T cells (%). As shown in Fig. 6, the levels of IL-10 and CD4^+^CD25^+^ Treg/CD4^+^ T cells (%) were significantly increased compared with pre-transplation and the correlation analysis of IL-10 and CD4^+^CD25^+^ Treg/CD4^+^ T cells (%) were positively correlated. Thus, the IL-10 and CD4^+^CD25^+^ Treg cells are interrelated and interact in antirejection efforts. Therefore, intramuscular injections of *in vitro* rhIL-10 post-transplantation led to an increase in IL-10 and CD4^+^CD25^+^ Treg cells, and this may be the mechanism by which IL-10 inhibits allograft rejection.

## Discussion

IL-10 is a cytokine synthesis inhibitory factor, with a molecular weight of 17 kDa and contains 160 amino acid residues and two intramolecular disulfide bonds. The human IL-10 gene is located on the long arm of the chromosome 1q31–32 region. A number of human cells produce IL-10, including the specific T cell subsets, Th2, Tr1, Tc2, macrophages, monocytes, mast cells, liver cells and tumor cells (9). IL-10 is capable of inhibiting the cellular immune response and inflammation through the following channels. By decreasing the level of MHC-II, costimulatory molecules, including CD86 and adhesion molecules on the cell surface, IL-10 inhibits the antigen-presenting ability of monocytes and macrophages. Inhibition of IL-2, produced by mononuclear cells, is the basic method in T cell proliferation in the responses of specific cellular immunity. Inhibition of monocytes and macrophages produces inflammatory mediators, resulting in the reduction of IL-1, IL-8, IL-6, IFN-γ, TNF-β, granulocyte colony stimulating factor and granulocyte-macrophage colony stimulating factor when released (10). Previous studies have shown that local transfection of plasmid IL-10 in tissue and organs inhibits T cell activation and proliferation, induces T cell apoptosis, and reduces immunological rejection (11,12). In a lung transplantation model of ischemia-reperfusion injury, the receptor with transfected plasmid IL-10, exhibited lower IL-2, IFN-γ and TNF-α levels compared with the control group. Its pulmonary edema and function were improved in the control group (11). The present study provided evidence that IL-10 exhibits a protective effect in lung transplantation. The results indicated that the application of IL-10 in rabbit skin grafts may postpone the graft rejection and extend the survival time. The survival time of the combined group was longer than lower-dose IL-10 and low-dose CsA groups, suggesting that IL-10 has a synergistic effect with CsA. The IL-10 in rabbits increased following use of rhIL-10. Thus, rhIL-10, as a foreign antigen, is hypothesized to activate T cells, macrophages and Tregs, enhancing the function of IL-10 secretion, resulting in significantly increased postoperative levels of IL-10. IL-10 through the effects of autocrine, paracrine and endocrine systems inhibits the immune responses.

The CD4^+^CD25^+^ Tregs comprise ~5–10% of CD4^+^ T cells in normal human or mouse peripheral blood and are an important group of immune cells, which have inhibitory functions. CD4^+^CD25^+^ may negatively regulate the activation and proliferation of T cells. They are important in inducing and maintaining peripheral tolerance (8). Tregs may be divided into natural Tregs, which originate in the thymic medulla and induce regulatory T cells and adaptive Tregs, which arise from CD4^+^ T cells when infection, transplantation or cancer occurs. IL-10 and TGF-β may promote effector T cells into Tregs. Clinical studies confirmed that CD4^+^CD25^+^ Tregs are associated with transplantation tolerance. In lung transplant receptors, the number of Tregs in symptoms of chronic rejection is lower compared with patients without clinical symptoms and healthy controls. Following kidney transplantation, the number of Tregs in the long-term survival in patients is significantly increased. Tregs inhibit the donor CD4^+^ and CD8^+^ T cells, leading to T cell loss of function and may reduce the expression of the molecule at the antigen presenting cell surface, which is required for T cell activation; thus, reducing graft versus host disease (GVHD) (13). The Treg cells could be induced by antigen and IL-10, and in turn IL-10 secretion by Treg cells inhibited the activation and proliferation of T cells (14). The generation of Tregs also inhibits the antigen-specific immune response and positive lower immune responses. T cells have no response and Tregs are increased by IL-10 to maintain the antigen specificity of peripheral T cell tolerance (15). A previous study showed that an increase occurred when IL-10 was added to Tregs *in vitro* and the inhibition of Tregs following amplification was similar to that of natural Tregs (16). The aforementioned studies suggest that the inhibition of IL-10 immune function is closely associated with Tregs. In the current study, Tregs were at a lower level in the peripheral blood prior to skin graft and the levels were significantly increased following transplantation of skin. Allograft skin, as a foreign antigen*,* stimulates the immune system, resulting in lymphocyte activation and proliferation and a further increase in the Treg levels, the Tregs were present in higher levels and exhibited a longer survival time. The Treg levels in the IL-10 group were higher compared with the CsA and control groups following surgery and the levels in the CsA group were lower than in the control group. Thus, this indicates that rhIL-10 is conducive to Treg proliferation and CsA inhibits Tregs. T cell activation may be affected by CsA through blocking the transcription of IL-2. IL-2, as a signal transmitter, is important for the maintenance of Tregs. By blocking the transcription of IL-2, the activation and function of Tregs may be altered. Therefore, CsA not only reduced Treg proliferation, but also inhibited its function continuously. However, in the current study, IL-10 inhibited the rejection reaction in the grafts but not Tregs, compared with increased normal levels of Tregs, indicating a mechanism by which IL-10 inhibits allograft rejection.

TGF-β may reduce the immune response in a number of component elements; thus, it may exhibit potential immunosuppressant actions to be used for preventing graft rejection. It was reported that in organ transplant receptors *in vivo*, the expression of TGF-β is associated with graft versus host disease (GVHD) and following transplantation, the patients with highly expressed TGF-β, exhibited significantly reduced GVHD (17). The membrane-type TGF-β is activated when the foreign antigen activates Tregs, thus, the levels of TGF-β in the peripheral blood increase and inhibit the immune response (18). In the current study, the increased levels of TGF-β may be associated with increasing Tregs. The level of TGF-β did not alter the role of IL-10 and CsA and thus, it may be hypothesized that the mechanism of intramuscular injection of IL-10 to the inhibited immune response is not associated with TGF-β.

Rejection reaction is problematic for clinicians and immunologists and constantly causes difficulty in organ transplantation application. Although rejection has been relieved by tissue typing and immunosuppressant application e.g., CsA, the issue of rejection has not been entirely overcome. It is urgent to develop novel effective anti-rejection drugs. A number of studies hypothesize that IL-10 may become a novel immunosuppressant for clinical application due to its immunosuppressive activity. The current results show that IL-10 may inhibit rabbit skin allograft rejection and its mechanism may be associated with Tregs and IL-10.

## Abbreviations

rhIL-10: recombinant human interleukin-10
FCM: flow cytometry
ELISA: enzyme-linked immunosorbent assay
CsA: cyclosporin A
